# Tissue-Specific Influence of Lamin A Mutations on Notch Signaling and Osteogenic Phenotype of Primary Human Mesenchymal Cells

**DOI:** 10.3390/cells8030266

**Published:** 2019-03-21

**Authors:** Kseniya Perepelina, Polina Klauzen, Anna Kostareva, Anna Malashicheva

**Affiliations:** 1 Almazov National Medical Research Centre, 2 Akkuratova Str., St-Petersburg 197341, Russia; kseniya.perepelina@mail.ru (K.P.); polina.klauzen@gmail.com (P.K.); akostareva@hotmail.com (A.K.); 2 St-Petersburg State University, 7-9, Universitetskaya nab., St-Petersburg 199034, Russia; 3 Institute of Cytology, Russian Academy of Sciences, 4 Tikhoretsky Ave., St-Petersburg 194064, Russia

**Keywords:** lamin A, Notch signaling, osteogenic differentiation

## Abstract

Lamin A is involved in many cellular functions due to its ability to bind chromatin and transcription factors and affect their properties. Mutations of *LMNA* gene encoding lamin A affect the differentiation capacity of stem cells, but the mechanisms of this influence remain largely unclear. We and others have reported recently an interaction of lamin A with Notch pathway, which is among the main developmental regulators of cellular identity. The aim of this study was to explore the influence of *LMNA* mutations on the proosteogenic response of human cells of mesenchymal origin and to further explore the interaction of LMNA with Notch pathway. Mutations R527C and R471C in *LMNA* are associated with mandibuloacral dysplasia type A, a highly penetrant disease with a variety of abnormalities involving bone development. We used lentiviral constructs bearing mutations R527C and R471C and explored its influence on proosteogenic phenotype expression and Notch pathway activity in four types of human cells: umbilical vein endothelial cells (HUVEC), cardiac mesenchymal cells (HCMC), aortic smooth muscle cells (HASMC), and aortic valve interstitial cells (HAVIC). The proosteogenic response of the cells was induced by the addition of either LPS or specific effectors of osteogenic differentiation to the culture medium; phenotype was estimated by the expression of osteogenic markers by qPCR; activation of Notch was assessed by expression of Notch-related and Notch-responsive genes by qPCR and by activation of a luciferase CSL-reporter construct. Overall, we observed different reactivity of all four cell lineages to the stimulation with either LPS or osteogenic factors. R527C had a stronger influence on the proosteogenic phenotype. We observed the inhibiting action of *LMNA* R527C on osteogenic differentiation in HCMC in the presence of activated Notch signaling, while *LMNA* R527C caused the activation of osteogenic differentiation in HAVIC in the presence of activated Notch signaling. Our results suggest that the effect of a *LMNA* mutation is strongly dependent not only on a specific mutation itself, but also might be influenced by the intrinsic molecular context of a cell lineage.

## 1. Introduction

Nuclear lamins are the main proteins of the nuclear envelope and provide the strength to the nuclear membrane, as well as the interaction of extra-nuclear structures with components of the nuclear matrix. Recently, it became clear that lamins play not only a structural, but also a regulatory role in a cell. Disruption or mutation of the *LMNA* gene is associated with a disease called laminopathy. The most known pathological form of lamin A—progerin—causes a rare premature aging syndrome, or progeria. At the same time, point mutations of the *LMNA* gene encoding lamin A are more frequent and lead to diseases, in which various tissues of mesenchymal origin are damaged. Mutations are often tissue-specific, that is, certain mutations lead to the appearance of a single disease phenotype with a muscle, skeletal or adipose tissue being primarily involved. Dysfunction of the cardiovascular system is a common sign for many laminopathies and is considered as the leading clinical sign in lamin-associated cardiomyopathies and myodystrophies [1,2,3,4,5].

The studies focusing on the cells of cardiovascular origin are still rare and mechanisms of cardiac pathologies associated with mutations in *LMNA* are not clear. It has been shown that changes in the processing of lamina may be involved in atherosclerotic processes during aging. Pre-lamin A could accumulate in the arterial wall and colocalize with degenerating smooth muscle cells in atherosclerotic plaques [6,7]. Progerin also causes defects in smooth muscle cells (SMC) [8]. Lamin A has been shown to be involved in the regulation of proliferation and apoptosis of endothelial cells [9]. The accumulation of pre-lamin A in endothelial cells causes premature aging and functional impairment of the vascular wall in general [10]. In smooth muscle cells, over-expressing mutant lamin A increased oxidative stress, inflammation and calcification [11]. 

How lamins regulate gene expression and cell differentiation remains unclear. Lamins directly bind to DNA, chromatin, nucleosomes and histones, but the physiological relevance of these interactions is still not certain [12]. Long heterochromatic domains associated with lamins have been identified and named lamina-associated domains (LADs) [13]. Anchoring genes to the lamina correlate with tissue-specific gene repression leading to the concept that tethering of genomic regions to the lamina is required for stable repression of genes during differentiation [13,14,15]. Uncovering the mechanisms of the tissue-specific effect of lamin A mutations is, therefore, of key importance for understanding the target organ and tissue damage linked to a particular lamin mutant variant. 

One of the proposed mechanisms for the realization of the lamin pathological effect is a specific alteration of a particular cellular signaling system. We have recently shown that lamin A interacts with Notch signaling, influencing cellular fate and differentiation, and point mutation in *LMNA* could affect this interaction [16]. Lamin A-Notch interaction can be realized both through chromatin regulatory mechanism and through direct structural interactions, for example through emerin-dependent suppression of Notch signaling [17,18,19,20]. Involvement of lamins in regulating Notch signaling has also been shown for progerin [21]. 

Proosteogenic phenotype is one of the “default” cellular phenotypes that cells of mesenchymal origin could easily acquire at pathological state such as vascular and valvular calcification, atherogenic transformation, aging, and others. Notch is an important regulator of the osteogenic state of cells and is implicated in various stages of osteogenesis [22]. 

Mandibuloacral dysplasia type A (MADA) is an extremely rare autosomal recessive genetic disorder caused by the mutation of the *LMNA* gene. MADA is characterized by dysmorphic craniofacial and skeletal features, lipodystrophy, and metabolic complications as a consequence of severe alteration of cellular osteogenic differentiation and calcification processes [13,23,24,25,26,27,28]. During the study of this disease, the following point mutations associated with amino acid substitutions were found: R527H, R527C and A529V [25]. 

The aim of this study was to explore the influence of *LMNA* mutations on the proosteogenic response of human cells of mesenchymal/cardiovascular origin and to further explore the interaction of LMNA with Notch pathway. To address this aim, we have chosen two *LMNA* mutations associated with MADA and compared LMNA-Notch interaction in four cell types of mesenchymal/cardiovascular origin—endothelial, smooth muscle, cardiac and valvular interstitial cells. We show that the effect of a *LMNA* mutation is strongly dependent on the cell type and thus is tissue-specific and might be reversed depending on cell type even inside mesenchymal lineages. 

## 2. Materials and Methods

### 2.1. Human Samples

The study was performed according to the Declaration of Helsinki and approval was obtained from the local Ethics Committee of Almazov Federal Medical Research Centre. Written informed consent was obtained from all subjects prior to tissue biopsy. 

### 2.2. Cell Culture

Human and rat cardiac mesenchymal cells (HCMC and RCMC, correspondingly) from myocardial tissue were isolated and put in in vitro culture and induced to differentiation as previously described [29]. Briefly, the tissue was dissected in small clamps and digested by collagenase II (Worthington) in PBS (1 mg/mL) for 2 h at 37 °C. Then the suspension was passed through the 40 mkm strainer, centrifuged at 300× *g* for 5 min and cells were put in growth medium (22.5% EGM-2 (Lonza, Walkersville, MD, USA), 67.5% M199, 10% fetal bovine serum (Hyclone), 1× nonessential amino, 50 units/mL penicillin and 50 µg/mL streptomycin (Thermo Fisher Scientific, Waltham, MA, USA), seeded on 0.1% gelatine-coated 96-well plate, and clonally expanded. After this, the cells were expanded for 1–2 weeks in Petri dishes (Corning, NY, USA) in growth medium, at 37 °C and 5% CO_2_. CMC immunophenotype was verified with flow cytometer GuavaEasyCyte6 (Millipore, Billerica, MA, USA) using CD33, CD45, CD117, CD90, CD105, CD73, CD146, CD56 monoclonal antibodies (BD, USA) as previously described [16].

To obtain human aortic smooth muscle cells (HASMC), cultures the cells were isolated from the aortic wall by collagenase digestion as described previously [30]. HASMC were cultured in growth medium containing DMEM (Invitrogen, Waltham, MA, USA) supplemented with 20% fetal bovine serum (FBS) (Invitrogen, Waltham, MA, USA), 2 mM L-glutamine, sodium pyruvate, and penicillin/streptomycin (100 mg/L) (Invitrogen, Waltham, MA, USA). The cells were used in experiments at passages 2–7.

Human aortic valve interstitial cells (HAVIC) were isolated from aortic valves harvested during aortic valve surgery at the National Almazov Research Centre by collagenase treatment as described previously [31]. Patients with known infective endocarditis and rheumatic disease were excluded from the study. HAVIC from normal aortic valves were isolated from healthy valves obtained from explanted hearts from recipients of heart transplantation. HAVIC were cultured in growth medium containing DMEM (Invitrogen, Waltham, MA, USA) supplemented with 15% fetal bovine serum (FBS) (Invitrogen, Waltham, MA, USA) and 2 mM of L-glutamine, sodium pyruvate and penicillin/streptomycin (100 mg/l) (Invitrogen, Waltham, MA, USA). 

Human umbilical vein endothelial cells (HUVEC) were isolated from the umbilical vein by collagenase dissociation [32]. The vein was rinsed in PBS, filled by 0.1% collagenase solution (Collagenase, Type II) (Worthington Biochemical Corporation, Lakewood, NJ, USA) and incubated in PBS at 37 °C for 10 min. The suspension was centrifuged at 300× *g* for 5 min. The cell pellet was suspended and seeded on a 35-mm Petri dish covered with 0.2% gelatin in ECM (ScienCell, Logan, UT, USA). Primary cells between passages two and five were used for all experiments. All cultures were maintained in humidified 5% CO_2_ at 37 °C.

### 2.3. Lentiviral Constructs and Transduction

*LMNA* WT, *LMNA* R527C and *LMNA* R471C bearing lentiviral constructs were described in a previous work [26] as well as Notch-intracellular domain (NICD) lentiviral construct [33]. Lentiviral packaging plasmids were a gift of D.Trono (École Polytechnique Fédérale de Lausanne, Lausanne, Switzerland); pLVTHM was modified by the addition of the T7 tag and chloramphenicol resistance gene (cm), resulting in the pLVTHM-T7-cm vector. 12xCSL-luc construct was a kind gift of prof. U. Lendahl (Karolinska Institute, Stockholm, Sweeden). The reporter sequence was subcloned into pLVTHM-T7-cm vector.

### 2.4. Lentiviral Production and Transduction

Lentiviral production was performed as previously described [33]. In short, 100-mm dishes of nonconfluent 293 T cells were cotransfected with 15 μg pLVTHM-T7-NICD, 5.27 μg pMD2.G, and 9.73 μg packaging pCMV-dR8.74psPAX2 by the calcium-phosphate method. The next day, the medium was changed to the fresh one, and the cells were incubated for 24 h. Produced lentivirus was concentrated from supernatant by the ultracentrifugation method at 20,000 rpm for 2 h, resuspended in 1% BSA/PBS and frozen in aliquots at −80 °C. Concentrated viral particles were added 1 h after cells trypsinisation; 6 h later the culture medium was changed.

Transgene expression from lentiviruses was verified. For NICD-bearing, virus transduction of cells was verified by Western blotting for active Notch domain (NICD). For *LMNA*-bearing viruses, RCMC were transduced with viruses bearing human *LMNA* and then immunocytochemical staining with antibodies against human lamin A was performed. The effective rate of RCMC transduction was 40–60%.

### 2.5. Immunocytochemical Staining

The cells were grown on cover slides, then fixed for 10 min in 4% paraformaldehyde and treated by 0.1% Triton X-100/PBS for 3 min, then incubated in 1% BSA/PBS for 1 h. Then cells were incubated for 1 h with primary antibodies against lamin A (Leica, Wetzlar, Germany). Secondary antibodies conjugated with Alexa488 (Invitrogen, Waltham, MA, USA) were used. DAPI was used to visualize nuclei. Microphotographs were taken using AxioObserver Microscope (Zeiss, Oberkochen, Germany) at ×20 and ×40 magnification with AxioVision software.

### 2.6. Induction of Osteogenic Differentiation 

Intact cells and cells were transduced with virus carrying wild type *LMNA WT* or mutant *LMNA. LMNA R527C* and *LMNA R471C* were used in experiments. Osteogenic differentiation was induced in control and transduced cells by the addition of 50 mg/m ascorbic acid, 0.1 mM dexamethasone and 100 mM beta–glycerophosphate to the osteogenic medium (for HUVEC: Endothelial Cell Medium (ECM) (ScienCell); for HCMC: DMEM/F12 was supplemented with 2% horse serum, 1× nonessential amino, 1× ITS, 2 mM L-glutamine, and 100 units/mL penicillin/streptomycin; for HASMC: DMEM was supplemented with 5% FBS (HyClone), 2 mM L-glutamine, and 100 units/mL penicillin/streptomycin; for HAVIC: DMEM was supplemented with 15% FBS (HyClone), 2 mM L-glutamine, and 100 units/mL penicillin/streptomycin). All experiments were performed with three biological replications, i.e., three independent experimental procedures. The expression of osteogenic markers were estimated after 3 days of differentiation using qPCR.

### 2.7. qPCR

RNA from cultured cells was isolated using ExtractRNA (Eurogene, Moscow, Russia). Total RNA (1 μg) was reverse-transcribed with MMLV RT kit (Eurogen, Moscow, Russia). Real-time PCR was performed with 1 μL cDNA and SYBRGreen PCRMastermix (Eurogen, Moscow, Russia) or TaqMan in the Light Cycler system. The thermocycling conditions were as follows: 95 °C for 5 min, followed by 45 cycles at 95 °C for 15 s and 60 °C for 1 min. A final heating step of 65 °C to 95 °C was performed to obtain melting curves of the final PCR products. The corresponding gene expression level was normalized to GAPDH from the same samples. Changes in the target genes expression levels were calculated as fold differences using the comparative ΔΔCT method. Primer sequences for human *NOTCH1*, *NOTCH3, NOTCH4, HEY1*, *HES1*, *RUNX2, OPN*, *and COL1A1* are presented in Table 1. ATF4 expression was analyzed using HS00909569_g1 probe (Thermo Fisher Scientific, Waltham, MA, USA).

### 2.8. Promoter Activity Assay

Cells were transduced with lentivirus containing the 12xCSL-luc reporter described above. In the construct, the expression of the firefly luciferase gene is regulated by 12 CSL binding sites upstream of a minimal TK promoter and the level of CSL promoter activity indicates the transcriptional activation of a Notch pathway. Cells were lysed using Luciferase Assay System (Promega, Madison, USA) according to the manufacturer’s recommendations 48 h after transduction. Luciferase activity was measured with Synergy2 (BioTek, Winooski, Vermont USA). Samples were normalized by protein content using Pierce BCA Protein Assay Kit (Thermo Fisher Scientific, Waltham, MA USA). 

### 2.9. Statistics

Values are expressed as mean ± SD of triplicate experiments. Groups were compared using Student’s *t*-test. A value of *p* ≤ 0.05 was considered significant. Statistical analysis was performed by using R software (version 2.12.0; R Foundation for Statistical Computing, Vienna, Austria).

## 3. Results

### 3.1. LMNA R527C and LMNA R471C Causes Disruption of Lamin Organization in CMC

**Figure 1:**
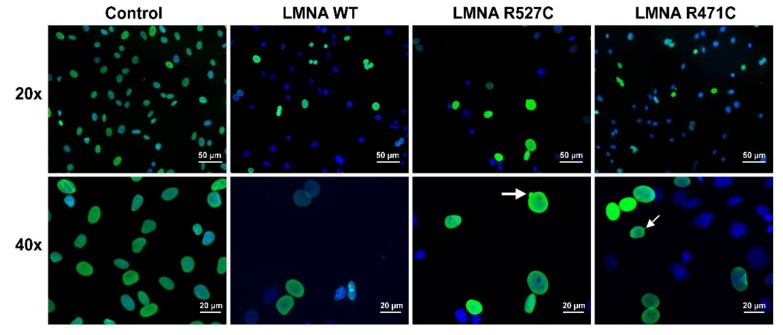
Verification of transgene LMNA expression in CMC. RCMC were infected with saturating concentrations of lentiviruses bearing human *LMNA* WT or *LMNA* R527C/R471*C* genes. The cells were stained with anti lamin A antibody recognizing only human lamin A. Upper row: 20× magnification; lower raw 40× magnification. HCMC were used as a control (Control) and give uniform nuclear staining of lamin A at the nuclear rim. Transduction of RCMC with lentivirus-bearing human *LMNA* WT shows nuclei with normal appearance and without any blebbing (LMNA WT). Transduction of RCMC with lentivirus bearing human *LMNA* R527L and *LMNA* R471C shows nuclear blebbing in some of the transduced cells as an effect of the mutation.

We analyzed the distribution of lamin A in cardiac mesenchymal cells (CMC) transduced with lentiviruses bearing wild type and mutant *LMNA*. For this, we transduced rat cardiac mesenchymal cells (RCMC) with lentiviruses containing human sequences of *LMNA* WT, *LMNA* R527C and *LMNA* R471C, and then stained the cells with an antibody specifically recognizing human lamin A and not recognizing rat lamin A (Figure 1). That allowed us to analyze exclusively the transgene expression of LMNA. In the control human non-transduced cells and in the cells transduced with *LMNA* WT, nuclei show lamin A at the nuclear rim with no alterations in nuclear morphology. The cells transduced with *LMNA* R527C and R471C show nuclear blebbing and alterations in nuclear morphology. 

### 3.2. The Impact of LMNA R527C and LMNA R471C on Osteogenic Markers in Human Mesenchymal Cells in the Presence of LPS

**Figure 2:**
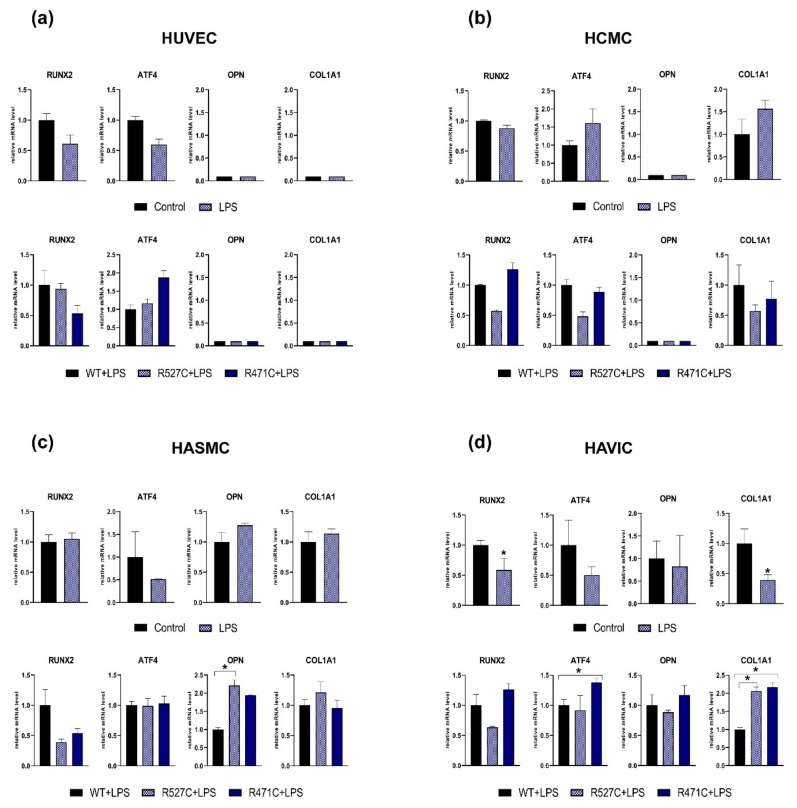
The effect of *LMNA* R527C and *LMNA* R471C mutations on the osteogenic marker expression in undifferentiated human mesenchymal cells with the presence of LPS. Upper panel for each type of cells HUVEC (**a**), HCMC (**b**), HASMC (**c**) and HAVIC (**d**) represents analysis of osteogenic marker expression by qPCR in undifferentiated cells without LPS (Control) and in the presence of LPS (LPS). Lower panel for each types of cells (a, b, c, d) represents the analysis of osteogenic marker expression by qPCR in cells transduced with lentivirus bearing either *LMNA* W*T* (WT) or *LMNA* R527C*/LMNA*R471C (R527C/R471C). The results are represented as mean ± SD; * *p* < 0.05.

Lipopolysaccharide (LPS) is widely used for its ability to mimic anti-inflammatory cellular response and has been shown to influence the proliferation and differentiation of mesenchymal stem cells [34]. To assess an impact of mutant LMNA on the expression level of osteogenic markers in the presence of LPS, we transduced cells with lentiviruses bearing *LMNA* WT or mutant *LMNA* (*LMNA* R527C/*LMNA* R471C) and added LPS to culture medium. We used four types of human cells of mesenchymal/cardiovascular origin: human umbilical vein endothelial cells (HUVEC), human cardiac mesenchymal cells (HCMC), human aortic smooth muscle cells (HASMC) and human aortic valve interstitial cells (HAVIC). We analyzed the expression of osteogenic markers *RUNX2, ATF4, OPN,* and *COL1A1* 3 days after transductions (Figure 2). We observed a significant decrease in the level of *RUNX2* and *COL1A1* in LPS-treated HAVIC compared to HAVIC without LPS (Control) in non-transduced cells HAVICs only. Next, we analyzed the effect of *LMNA* R527C and *LMNA* R471C compared to *LMNA* WT in LPS-treated cells. We detected a statistically significant increase of *OPN* and *COL1A1* expression levels caused by *LMNA* R527C mutation in HASMC and HAVIC, correspondingly. LMNA R471C caused a significant increase of *ATF4* and *COL1A1* in HAVIC when compared to LMNA WT. Thus, four types of mesenchymal cells had individual early responses to the LPS treatment.

### 3.3. The Impact of LMNA R527C and LMNA R471C on Osteogenic Differentiation in Human Mesenchymal Cells

**Figure 3:**
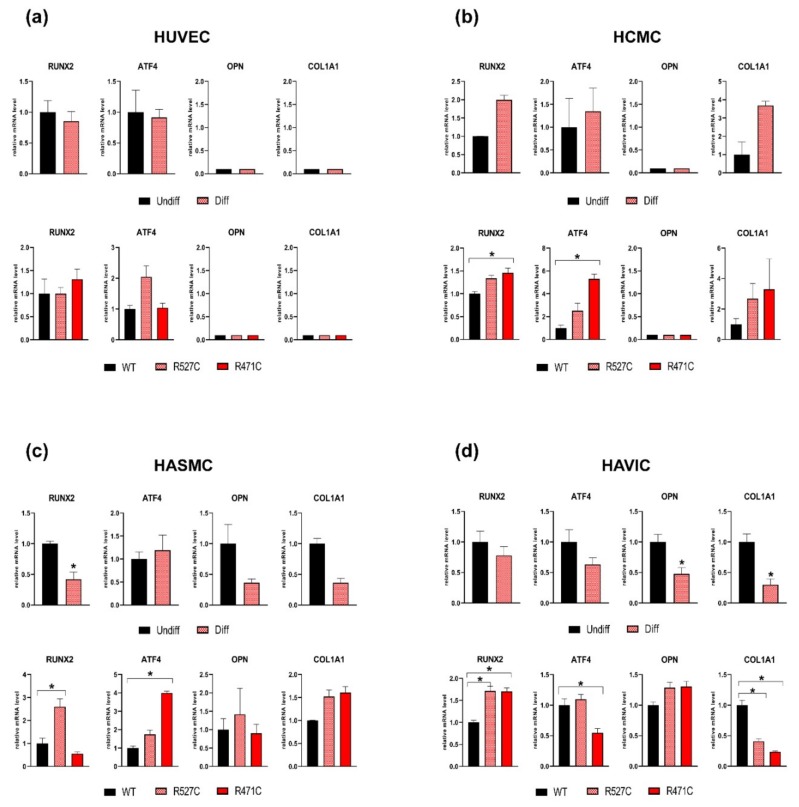
The effect of *LMNA* R527C and *LMNA* R471C mutations on the osteogenic differentiation process of human mesenchymal cells. Cells were differentiated in osteogenic direction for 3 days. Upper panel for each type of cell: HUVEC (**a**), HCMC (**b**), HASMC (**c**) and HAVIC (**d**) represent the analysis of osteogenic marker expression by qPCR in undifferentiated cells (Undiff) and after 3 days of osteogenic differentiation (Diff). Lower panel for each type of cell (a, b, c, d) represents analysis of osteogenic marker expression by qPCR in cells transduced with lentivirus bearing either *LMNA* WT (WT) or *LMNA* R527C*/LMNA*R471C (R527C/R471C). The results are represented as mean ± SD; * *p* < 0.05.

To explore proosteogenic capacity, we induced osteogenic differentiation in the cells by the addition of specific factors to growth medium. Osteogenic induction was analyzed by the early expression of specific osteogenic markers *RUNX2, ATF4, OPN* and *COL1A1* 3 days after the induction of differentiation (Figure 3). We observed a significant decrease of *RUNX2* expression level in differentiated HASMC and a decrease of *OPN, COL1A1* expression levels in differentiated HAVIC compared to undifferentiated cells. Next, we analyzed the effect of mutant LMNA on the expression of osteogenic genes in differentiating cells (Figure 3: lower panel a, b, c, d). We transduced cells with lentiviruses bearing *LMNA* WT or mutant *LMNA* (*LMNA* R527C and *LMNA* R471C) and induced osteogenic differentiation. Transduction with *LMN*A R471C caused a significant change in the expression level of osteogenic markers: increase of *RUNX2* and *ATF4* in HCMC; increase of *ATF4* in HASMC; increase of *RUNX2* and decrease of *ATF4* and *COL1A1* in HAVIC. Transduction with *LMNA* R527C caused a significant increase in the expression level of *RUNX2* in HASMC and HAVIC and a decrease in the expression level of *COL1A1* in HAVIC. In addition, we found a very low level of *OPN* and *COL1A1* genes expression in HUVEC and *OPN* gene expression in HCMC. These results show individual early response of each cell type on osteogenic induction and *LMNA* mutation. 

### 3.4. The Impact of LMNA R527C and LMNA R471C on Expression of Notch Target Genes in Human Mesenchymal Cells

**Figure 4:**
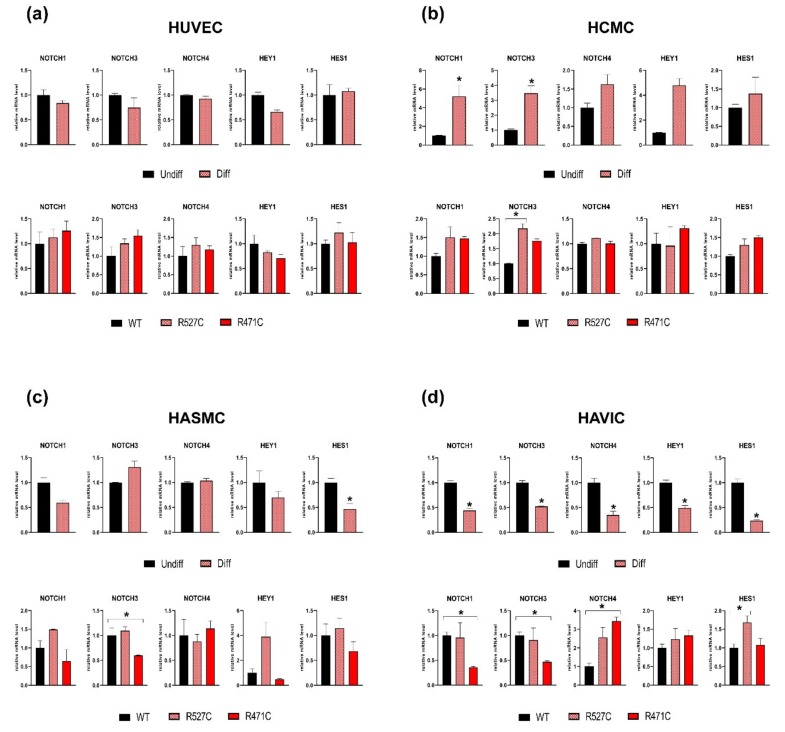
The effect of LMNA R527C and LMNA R471C mutations on the Notch pathway activity in human mesenchymal cells. Cells were differentiated in osteogenic direction for 3 days. Upper panel for each types of cells: HUVEC (**a**), HCMC (**b**), HASMC (**c**) and HAVIC (**d**) represent the analysis of Notch target gene expression by qPCR in undifferentiated cells (Undiff) and after 3 days of osteogenic differentiation (Diff). Lower panel for each types of cell (**a**, **b**, **c**, **d**) represents the analysis of Notch target gene expression by qPCR in cells transduced with lentivirus bearing either *LMNA WT* (WT) or *LMNA R527C/LMNAR471C* (R527C/R471C). The results are represented as mean ± SD; * *p* < 0.05.

To estimate the interaction of lamin A and Notch pathway, we analyzed the effect of mutant LMNA on the expression of Notch target genes in the four types of human mesenchymal cells: HUVEC, HCMC, HASMC and HAVIC (Figure 4). We transduced the cells with lentiviruses bearing *LMNA* WT or mutant *LMNA* (*LMNA* R527C/*LMN*A R471C) expression in each cell type. Induction of osteogenic differentiation caused a statistically significant increase in the expression of *NOTCH1* and *NOTCH3* in HCMC compared to undifferentiated cells. In contrast, the induction of osteogenic differentiation in HASMC and HAVIC caused a statistically significant decrease in the expression of *HES1* or *NOTCH1, NOTCH3, NOTCH4, HEY1,* and *HES1*, correspondingly. Next, we evaluated the effect of *LMNA* R527C and *LMNA* R471C mutations on Notch-related genes compared to *LMNA* WT. We detected a significant increase of the *NOTCH3* expression level in HCMC caused by *LMN*A R527C mutation when compared to *LMNA* WT. HASMC with LMNA R471C were marked by a significant decrease of *NOTCH3* expression level compared to LMNA WT. In addition, we demonstrated statistically significant changes of gene expression levels in HAVIC: decreasing *NOTCH1* and *NOTCH3* for cells with LMNA R471C, and increasing *NOTCH4* and *HES1* for cells witch LMNA R471C and LMNA R527C, correspondingly.

These data suggest that mutant lamin A counteracts with Notch signaling and confirms our earlier observation that the effect of mutations in *LMNA* are dependent on the cellular type and tissue.

### 3.5. LMNA R527C Mutation Has the Opposite Effect on HCMC and HAVIC in the Presence of Notch Activation

HCMC and HAVIC were selected for further analysis as they showed more pronounced effects of *LMN*A R527C mutations.

**Figure 5:**
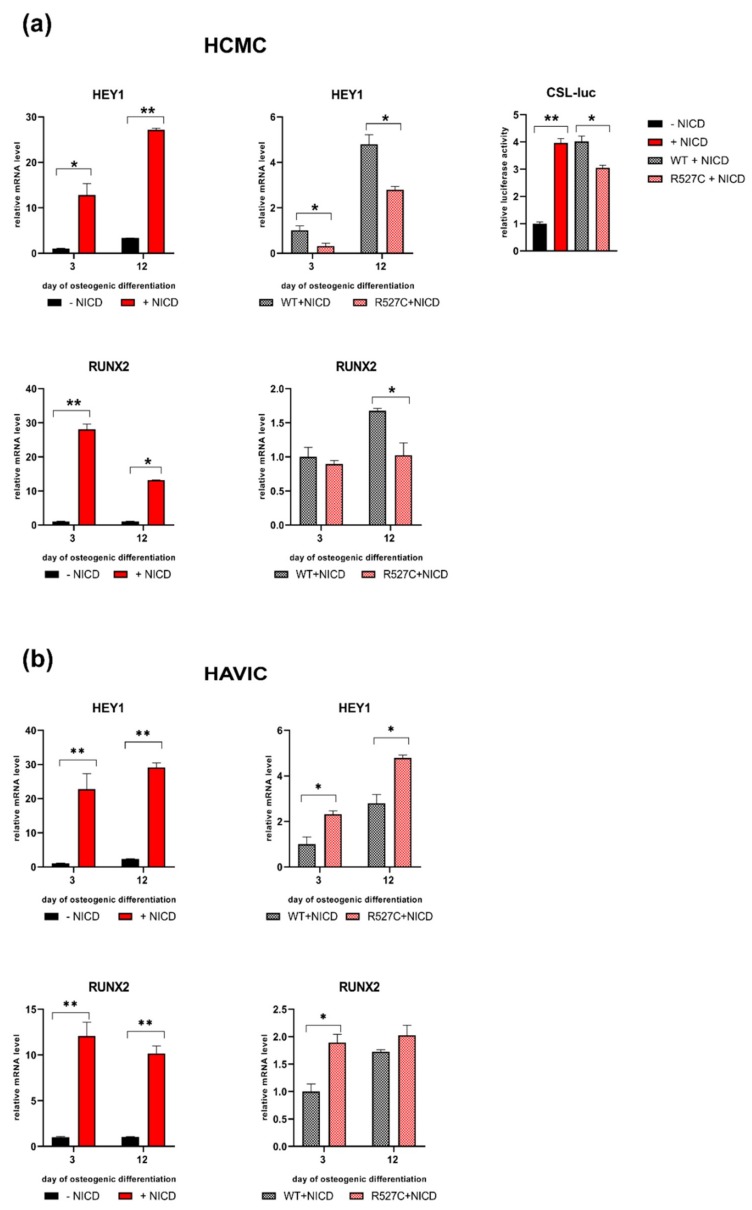
LMNA R527C mutation has the opposite effect on HCMC and HAVIC in the presence of Notch activation. (**a**) Upper panel: *LMNA R527C* affects Notch target gene expression in differentiated HCMC when Notch is activated. Osteogenic differentiation of HCMC was induced with/without Notch induction via transduction with lentivirus bearing Notch-intracellular domain (NICD) and in the presence of either *LMNA WT* (WT) or *LMNA R527C* (R527C). mRNA level of *HEY1* was measured by qPCR. Notch-dependent transcription was measured as luciferase activity of CSL-luc reporter. Lower panel: *LMNA R527C* affects differentiation ability of HCMC when Notch is activated. Osteogenic differentiation was induced with/without NICD and in the presence of either *LMNA WT* (WT) or *LMNA R527C* (R527C). mRNA level of osteogenic marker RUNX2 was measured by qPCR. The results are represented as mean ± SD, * *p* < 0.05, ** *p* < 0.01. (**b**) Upper panel: *LMNA R527C *affects Notch target gene expression in differentiated HAVIC when Notch is activated. Osteogenic differentiation of HAVIC was induced with/without NICD and in the presence of either *LMNA WT* (WT) or *LMNA R527C* (R527C). mRNA level of *HEY1* was measured by qPCR. Lower panel: *LMNA R527C* affects differentiation ability of HAVIC when Notch is activated. Osteogenic differentiation was induced with/without NICD and in the presence of either *LMNA WT* (WT) or *LMNA R527C* (R527C). mRNA level of osteogenic marker RUNX2 was measured by qPCR. The results are represented as mean ± SD, * *p* < 0.05, ** *p* < 0.01.

Notch signaling is known to be responsible for driving cells to various differentiated states including osteogenic lineage [35]. We sought to explore the effect of LMNA R527C on cellular phenotype in the presence of Notch activation in the course of osteogenic differentiation (3 and 12 days). We activated Notch signaling by transducing the cells with a Notch-intracellular domain (NICD) and induced osteogenic differentiation in HCMC and HAVIC (Figure 5). In the absence of transgenic LMNA, NICD substantially activated the expression of *HEY1,* indicating the activation of Notch signaling in both cell types at both time points (Figure 5a,b—upper panel). Further, we evaluated the influence of *LMNA* R527C mutation in the presence of Notch activation. Expression of *HEY1* decreased in HCMC bearing LMNA R527C compared to LMNA WT when Notch was activated. Correspondingly, luciferase activity of CSL reporter decreased in HCMC with LMNA R527C compared to LMNA WT, when Notch was activated. In contrast, HAVIC with LMNA R527C increased the expression level of *HEY1* compared to LMNA WT, when Notch was activated.

To study the effect of *LMNA* R527C mutation on osteogenic differentiation of HCMC and HAVIC in the presence of Notch activation, we applied the above-described method of activating the Notch pathway and transduction of cells by transgenic forms of *LMNA* (*LMNA* WT and *LMNA* R527C). Osteogenic induction was confirmed by the expression of a specific osteogenic marker *RUNX2* 3 and 12 days after the induction of differentiation. Figure 5 shows that the activation of Notch signaling leads to a significant increase in osteogenic potential in both cell types, which was observed by the *RUNX2* expression level. LMNA R527C caused a significant decrease in the expression level of *RUNX2* in HCMC 12 days after the induction of osteogenic differentiation. In contrast, there was an increase of the expression level of *RUNX2* in HAVIC 3 days after the induction of osteogenic differentiation.

Thus, we observed inhibiting action of LMNA R527C on osteogenic differentiation in HCMC in the presence of activated Notch signaling, while LMNA R527C caused the activation of osteogenic differentiation in HAVIC in the presence of activated Notch signaling.

**Figure 6:**
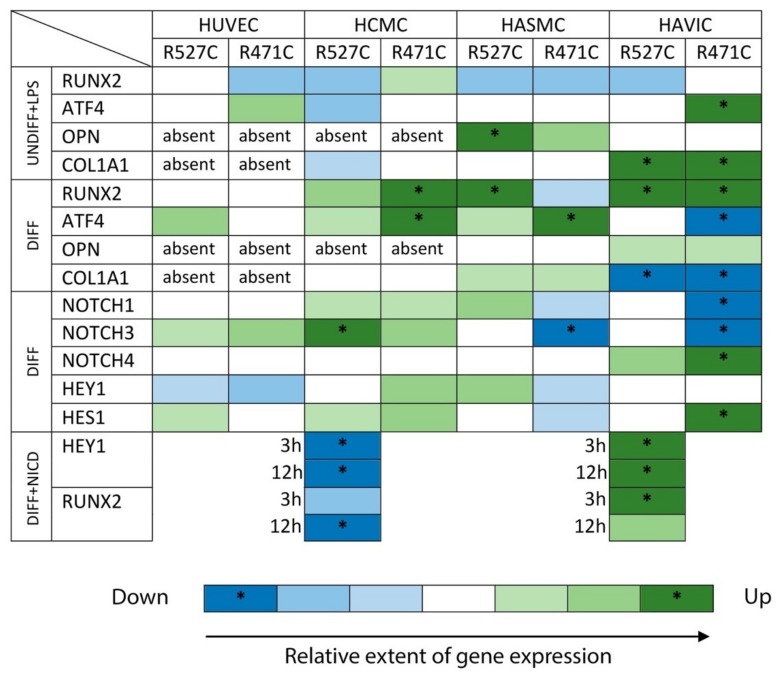
The extent of gene expression in different types of cells with LMNA mutation (R527C\R471C) in relation to LMNA WT (white). * *p* < 0.05. The levels of gene expression were measured by qPCR.

We summarized the data on gene expression in four cell lines in Figure 6. The data show that each line has an individual response to a *LMNA* mutation.

### 3.6. Analysis of Osteogenic Genes and Notch-Responsive Genes Expression in Different Lines of Undifferentiated Mesenchymal Cells 

**Figure 7:**
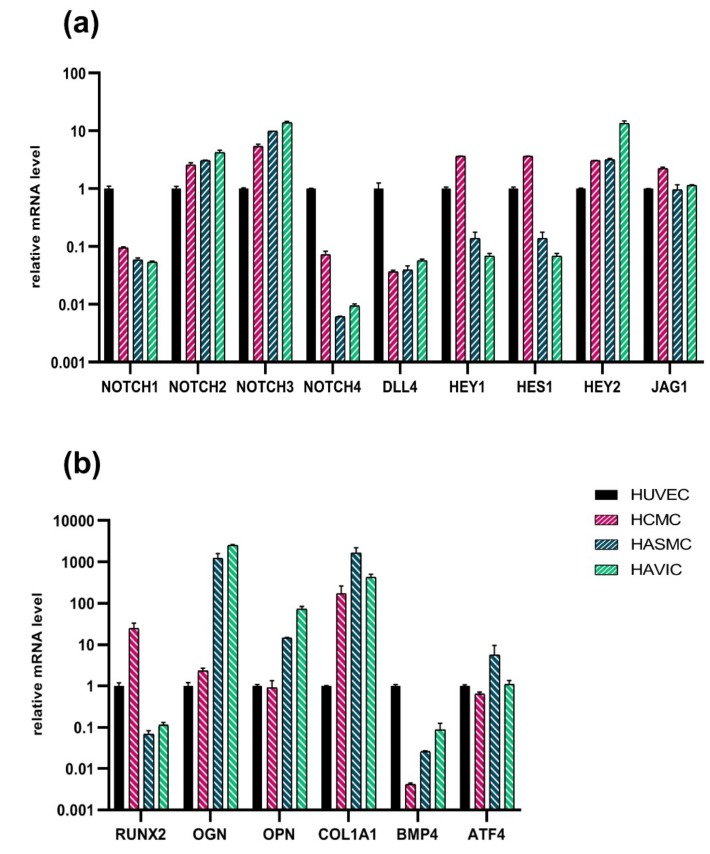
Comparison of Notch-related and proosteogenic gene expression between different cell lines of mesenchymal cells. (**a**) Analysis of Notch target gene expression by qPCR in undifferentiated cells. The results are represented as mean ± SD. A value of 1 was given to HUVEC mRNA levels for all genes (black). (**b**) Expression of osteogenic genes by qPCR in undifferentiated cells. Human umbilical vein endothelial cells (HUVEC), human cardiac mesenchymal cells (HCMC), human aortic smooth muscle cells (HASMC), and human aortic valve interstitial cells (HAVIC).

We asked what could be the reason for why the cells of the similar mesenchymal origin respond differently to the same condition. We analyzed the expression of Notch-related genes and proosteogenic genes and compared four mesenchymal lines used in the study by the level of expression of these genes (Figure 7). The expression level of a given gene was equated as 1 in HUVEC. Our results indicate that in spite of the common mesenchymal origin, the four lines have very different levels of expression of the same gene ranging up to 1000-fold. We suggest that this initial gene profile could also influence the effect of a given *LMNA* mutation in a given cell line.

## 4. Discussion

How lamins influence cellular fate remains one of the important questions of cellular biology. Understanding the regulating mechanisms, which involve lamins, is also important for the search for therapeutically relevant approaches to laminopathies caused by point mutations in the *LMNA* gene

In this study, we have analyzed the proosteogenic response of four human primary cell lineages in the presence/absence of specific *LMNA* mutations associated with bone tissue malformations. We show here that even the cells of close developmental origin, such as endothelial cells, cardiac mesenchymal cells, aortic smooth muscle cells, and aortic valve interstitial cells could have different consequences for the same conditions, such as specific proosteogenic treatment or specific *LMNA* mutation, in terms of their differentiation commitments. 

Previously, using human mesenchymal stem cells of adipose tissue (MSC), we have shown that *LMNA* mutations associated with various disease phenotypes bring about their effect in a mutation specific manner: Each mutation caused a specific influence on the differentiation and proliferation capacity of MSC [26]. This explained various mutation-dependent phenotypes seen in patients with laminopathies and also partially explained discrepancies seen in the literature concerning the influence of different mutations on cellular phenotype [36,37,38]. 

Direct binding of lamins to different regions of chromatin in the course of cellular differentiation led to the concept that differentiation might be dependent on the repression/activation of genes involved in this binding [12], however, specific pathways involved in these interactions are hardly known. Availability of genomic regions for activation by lineage-specific factors is regulated in part through dynamic chromatin-nuclear lamina interactions and the competence of a progenitor cell to respond to differentiation signals may depend upon coordinated movement of responding gene loci away from the nuclear periphery [39]. The data obtained in several laboratories suggest that, in the presence of *LMNA* mutations and, by extension, in all nuclear envelop disorders, there is an inappropriate association of heterochromatin with nuclear lamina upon differentiation [40,41].

In our previous work, we showed that *LMNA* R482L mutation associated with Dunnigan-type familial partial lipodystrophy contributes to the down regulation of Notch activation in undifferentiated and differentiated mesenchymal stem cells and decreases adipogenic differentiation when Notch is activated, thus supporting the hypothesis that lamin A interacts with Notch pathway [16]. In the present study, we show that lamin A mutations could have a critical effect on osteogenic differentiation depending on Notch activation and cell type. Notch is one of the key pathways ensuring development, differentiation and maintenance of adult tissues [42]. Notch is known to be involved in the regulation of osteogenic differentiation [35]. An implication of Notch in the interactions with lamins A has been shown for progerin [21]. In a recent report, emerin, a lamin binding partner, was shown to be able to directly interact with the Notch intracellular domain, thereby suppressing Notch activity [19].

Lamin A has been shown to influence osteoblast differentiation [43] and could constitute the determinant factor in the pathogenesis of both sarcopenia and osteopenia [27]. Several studies have described that progerin expression is associated with functional impairment in mesenchymal stem cells, reporting premature osteogenic differentiation [21,44]. Several *LMNA* mutations, either leading to a lipodystrophy or associated with signs of premature ageing, also triggered vascular smooth muscle cell senescence with osteoblastic transdifferentiation and calcification [11,45].

We observed various responses of different cell lineages to similar treatments stimulating proosteogenic phenotype and to similar alterations of genetic background such as the introduction of *LMNA* R527C or *LMNA* R471C mutations. A recent study used three cell lines: normal human dermal fibroblast, HeLa and HEK 293 and suggested that less differentiated embryonic cells are very sensitive to lamin A imbalance, and its upregulation disturbs lamin C, which may influence gene expression and many regulatory pathways [46].

Evidences exist that the expression of disease-causing lamin A mutations can drastically alter interactions with chromatin [41]. For example, it has been shown that the lipodystrophic lamin A R482W mutation alters 3D genome conformation [47]. Such findings indicate that lamin A is implicated in the developmental regulation of gene expression by promoting relocalization of loci towards the repressive nuclear periphery, restricting promoter–enhancer interactions and scaffolding epigenetic modifying complexes at relevant loci. All these findings suggest that lamin A mutations could differently alter the cell fate of different cell lineages [41].

The main conclusion that we draw from our study is that the action of lamin A is strongly dependent on the intrinsic molecular context. We observed a broad range in expression level of Notch-related and proosteogenic genes between the different cellular types used in the study. We suggest that this variability in gene expression level and balance among different signaling pathways could also contribute to spacial regulation of lamin interaction with chromatin and define its regulatory role in cellular fate. A comparison of four cellular models gives clues to understanding the variable and the still cryptic role of lamin A in driving cellular commitments to differentiation into a specific lineage.

Outcomes of this cross-talk deserve further research for a more detailed understanding of the role of A-type lamins in cellular differentiation as well as in pathology.

## Figures and Tables

**Table 1:** Primer sequences used for to amplify Notch target genes and osteogenic differentiation markers by qPCR.

Gene	Primer (5′→3′)
*GAPDH*	F: AATGAAGGGGTCATTGATGG
	R: AAGGTGAAGGTCGGAGTCAA
*NOTCH1*	F: GTCAACGCCGTAGATGACC
	R: TTGTTAGCCCCGTTCTTCAG
*NOTCH2*	F: ATGGTGGCAGAACTGATCAAC
	R: TTGGCAAAATGGTCTAACAGG
*NOTCH3*	F: GGAGCCAATAAGGACATGCAGGAT
	R: GGCAAAGTGGTCCAACAGCAGC
*NOTCH4*	F: GTTGTGACAGGGTTGGGACT
	R: CAGCCCAGTGGGTATCTCTG
*DLL4*	F: AGGCCTGTTTTGTGACCAAG
	R: CTCCAGCTCACAGTCCACAC
*JAG1*	F: TGCCAAGTGCCAGGAAGT
	R: GCCCCATCTGGTATCACACT
*HEY1*	F: TGGATCACCTGAAAATGCTG
	R: CGAAATCCCAAACTCCGATA
*HES1*	F: AGCACAGAAAGTCATCAAAG
	R: AGGTGCTTCACTGTCATTTC
*RUNX2*	F: TGGATCACCTGAAAATGCTG
	R: CGAAATCCCAACTCCGATA
*BMP4*	F: AGCACTGGTCTTGAGTATCCTG
	R: GCAGAGTTTTCACTGGTCCC
*COL1A1*	F: GACCTAAAGGTGCTGCTGGAG
	R: CTTGTTCACCTCTCTCGCCA
*OPN*	F: TCACCTGTGCCATACCAGTTAAA
	R: TGGGTATTTGTTGTAAAGCTGCTT
*OGN*	F: GGCAATAACACCATTACCTCCC
	R: AGGGTGGTACAGCATCAATGT
*ATF4 (TaqMan)*	HS00909569_g1
